# Management of gingival hyperpigmentation using microneedling with ascorbic acid vs. scalpel technique: A comparative split-mouth study

**DOI:** 10.34172/joddd.025.41707

**Published:** 2025-03-31

**Authors:** Anshika Maheshwari, Shruti Tandon, Arundeep Kaur Lamba, Farrukh Faraz, Nasreen Ansari

**Affiliations:** ^1^Department of Periodontology, Maulana Azad Institute of Dental Sciences, New Delhi, India; ^2^Maulana Azad Institute of Dental Sciences, New Delhi, India

**Keywords:** Ascorbic acid, Gingiva, Hyperpigmentation, Melanin, Minimally invasive surgical procedures, Randomization

## Abstract

**Background.:**

In enhancing smile aesthetics, managing gingival hyperpigmentation has become increasingly important. Gingival hyperpigmentation can often affect individuals psychologically, prompting many individuals to seek treatment. Microneedling (MN) is emerging as a popular solution for minimizing gingival hyperpigmentation through a minimally invasive approach. In contrast, the scalpel technique is an established surgical intervention to remove pigmented tissue directly. This study investigated the effectiveness of these two techniques in terms of clinical and patient-related outcomes.

**Methods.:**

This randomized control clinical study included 20 subjects with hyperpigmentation in both maxillary and mandibular arches. One arch of each subject was randomly allocated to MN using the coin toss method, and the other arch was assigned to the scalpel technique. Intraoperative bleeding, postoperative pain scores, wound healing index, Dummett oral pigmentation index (DOPI), and Hedin melanin index (HMI) were evaluated.

**Results.:**

MN showed significantly better results in terms of pain, bleeding scores, DOPI, and HMI (*P*<0.01). In contrast, the scalpel technique revealed significantly superior results in terms of wound healing.

**Conclusion.:**

Being a gold standard, the scalpel technique offers precision but is accompanied by more bleeding and less patient compliance due to its invasiveness. Conversely, the MN technique emerges as an advanced technique, demonstrating less pain and bleeding, thus enhancing patient comfort and overall satisfaction, aligning with the evolving preferences for minimally invasive dental procedures.

## Introduction

 An esthetic smile reflects self-esteem encompassing both psychological and physical dimensions. The health and appearance of the gums are essential elements contributing to an attractive smile. Typically, the color of the gingiva appears pink. However, some individuals present with diffuse deep brownish-black discoloration or black patches and striae or strands within the gums, known as gingival hyperpigmentation. This condition is often considered esthetically undesirable by certain people, particularly those with high smile lines and excessive gingival display. Factors like blood vessels, level of keratinization, epithelium width, and melanin pigmentation influence the color of the gingiva.^1^

 Gingival pigmentation may be caused by various by-products of physiological processes, including melanin, melanoid, reduced iron, and bilirubin. The quantity of melanin produced in an individual is primarily determined by genetics.^2^ Other factors may contribute to an increase in the production of melanin, such as hormones, smoking, trauma, radiation, and medications like minocycline, antimalarial drugs, anti-adrenocorticotropic hormone medications, and contraceptives.^3^

 Gingival depigmentation is a periodontal plastic procedure that involves surgical removal of gingival epithelium along with a layer of the underlying connective tissue, allowing the denuded connective tissue to heal by secondary intention. The new epithelium that forms is devoid of melanin pigmentation. Numerous methods have been established for gingival depigmentation, including scalpel scraping, bur abrasion, electrosurgery, cryotherapy, and laser treatments.^4,5^ Depigmentation with a scalpel remains the gold standard. However, there are several disadvantages associated, such as fear of surgical blades, pain, bleeding, healing by secondary intention, and an open wound site.^6^ The choice of technique should primarily consider the dentist’s clinical expertise and the patient’s preferences.^5,7^

 Microneedling (MN) technique is a minimally invasive surgical procedure known as collagen induction therapy involving repetitive tissue punctures. MN has gained substantial use in dermatology in recent years due to its effectiveness, simplicity, cost-effectiveness, good tolerance, and cosmetic and therapeutic benefits.^8^ MN operates by creating micro-conduits that separate cells rather than cutting through them, thus enhancing epithelium permeability and increasing blood flow to the epidermis. This process aids in penetrating topical medications through the stratum corneum layer. Additionally, it triggers the production of growth factors that promote collagen and elastin regeneration.^8^

 Ascorbic acid (AA) is a water-soluble antioxidant and an essential nutrient for collagen synthesis. It also plays a role in immunomodulation and the reduction of hyperpigmented spots by interacting with copper ions at the tyrosinase active sites, inhibiting the activity of the enzyme tyrosinase and subsequently reducing melanin pigmentation.^9^

 In the present study, MN with AA was used to treat gingival hyperpigmentation, and the results were compared with those of the scalpel technique. Several parameters were assessed, including intraoperative bleeding, pain scores one day postoperatively, wound healing at seven days, and the intensity and distribution of gingival pigmentation at baseline, one month, and six months.

## Methods

**Figure 1:**
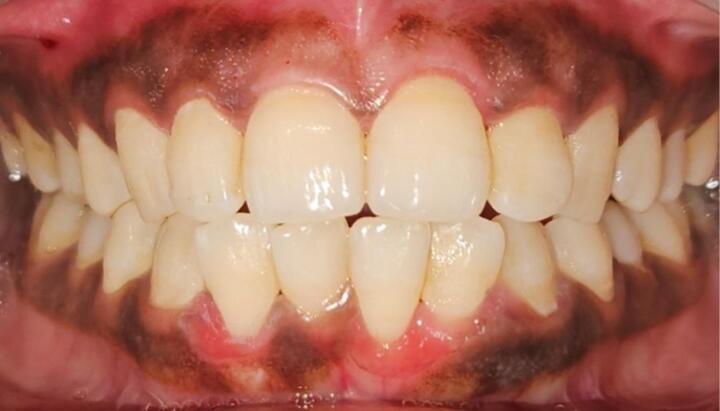
Preoperative picture of moderate gingival pigmentation

 After obtaining approval from the Institutional Ethics Committee, a randomized control clinical study was carried out in the Department of Periodontics, Maulana Azad Institute of Dental Sciences. Twenty-eight subjects with hyperpigmentation in both maxillary and mandibular arches, having healthy periodontium, were screened (Figure 1). Out of 28 subjects, 20 met the inclusion criteria and were included in the study. Randomization was done using the coin toss method to allocate technique 1 (scalpel method) or technique 2 (depigmentation with MN and AA) for different arches in each patient.

 Comprehensive extraoral and intraoral examinations were conducted, evaluating extraoral skin pigmentation to correlate it with intraoral pigmentation. The assessment included perioral pigmentation and detailed observation of the shape, color, surface, and borders of gingival melanin pigmentation.

 Inclusion criteria consisted of systemically healthy subjects with an age range of 20‒40 years having a Dummett oral pigmentation score of 2 or 3 (medium to deep brown gingival color) and Hedin melanin index (HMI) score of 3 or 4 (presence of a band of pigmented gingiva). Exclusion criteria were pregnancy, breastfeeding, systemic diseases related or unrelated to gingival melanin pigmentation, malignancy, medications, uncontrolled diabetes, and adverse reactions to AA. Twenty subjects meeting the inclusion criteria and signing informed consent underwent the gingival depigmentation procedure.

###  Parameters recorded

 The following criteria were assessed preoperatively, intraoperatively, and postoperatively at 1 and 6 months in the study groups by one examiner.

A. The bleeding score by Pavlic et al^10^ was assessed during the surgery, and the following scores were given according to the criteria suggested:

Score 1: No bleeding

 Score 2: A wound with a slight oozing of blood

 Score 3: Wound with moderate bleeding that can be controlled with a pressure pack

 Score 4: A wound with severe bleeding

B. The pain was assessed using a visual analog scale (VAS) on day 1 postoperatively, represented by a 10-cm horizontal line, where one endpoint denotes the absence of pain (score: 0) and the opposite end denotes severe pain (score: 10). Patients’ pain scores were diligently solicited as follows: 0 indicated no pain, 1–3 indicated mild pain, 4–6 indicated moderate pain, and 7–10 indicated severe pain.^11^
C. Wound healing was assessed on the 7th day postoperatively based on the healing index of Landry.^12^

Very poor: Tissue color: ≥ 50% of gingiva red, response to palpation, bleeding, granulation tissue present, incision margin: not epithelialized, with loss of epithelium beyond the incision margin, suppuration present

 Poor: Tissue color: ≥ 50% of gingiva red, response to palpation, bleeding granulation tissue present, incision margin: not epithelialized, with loss of epithelium beyond incision margin, suppuration present.

 Good: Tissue color: ≥ 50% of gingiva red, response to palpation, bleeding, granulation tissue present, incision margin: not epithelialized, with connective tissue exposed.

 Very good: Tissue color: ≥ 25% and 50% of gingiva red, response to palpation, no bleeding, no granulation tissue, incision margin: no connective tissue exposed.

 Excellent:Tissue color: < 25% of gingiva red, response to palpation, no bleeding, no granulation tissue, incision margin: no connective tissue exposed tissue color: all tissues pink.

D. The Dummett oral pigmentation index (DOPI)^13^ was used to assess the pigmentation intensity of the patients at baseline,1 month, and 6 months, with the following scoring system:

Grade 0: pink gingiva

 Grade 1: mild light brown gingival color

 Grade 2: medium brown gingival color or mixed brown and pink

 Grade 3: deep brown/blue-black gingival color

E. The HMI^14^ was used to assess the distribution of pigmentation at baseline,1 month, and 6 months in the gingiva, with the following scoring system:

Score 0: no gingival pigmentation

 Score 1: one or two isolated interdental papillae units that are pigmented

 Score 2: more than three distinct interdental papillary units that are pigmented

 Score 3: short continuous band of pigmented gingiva

 Score 4: one wideband continuous pigmented gingiva between the canines

###  Surgical procedures

####  Conventional scalpel technique

**Figure 2:**
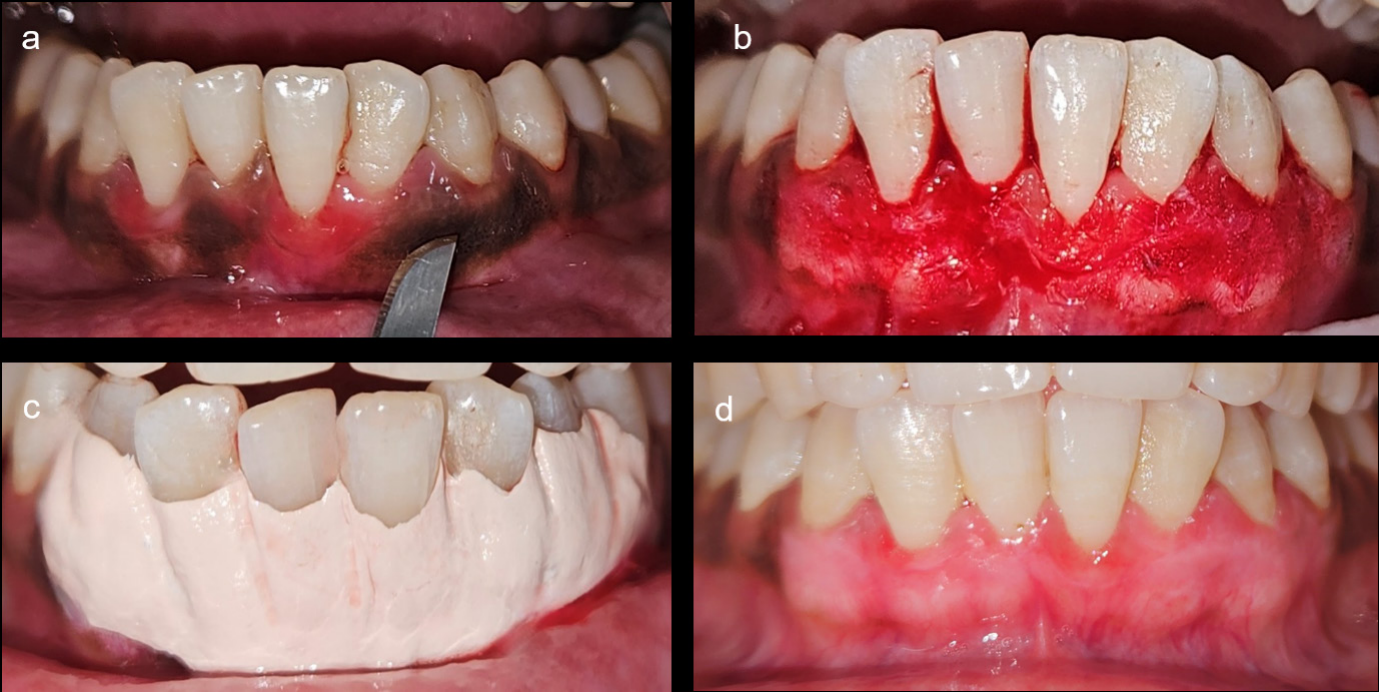
Depigmentation using scalpel technique. A) Epithelium, along with a thin layer of connective tissue, is removed using a #15 blade. B) Immediate postoperative view. C) Coe-pack placed. D) Postoperative view after 1 month

 In the control group, the scalpel technique was used. The region was anesthetized using 2% lignocaine hydrochloride. The entire gingival epithelium and some connective tissue were removed using a #15 surgical blade extending from the mucogingival junction to the marginal gingiva. Hemorrhage during the surgery was managed through direct pressure using sterile gauze. After depigmentation, the site was covered with a periodontal dressing (Coe-Pack) (Figure 2).

####  Depigmentation with microneedling and ascorbic acid

**Figure 3:**
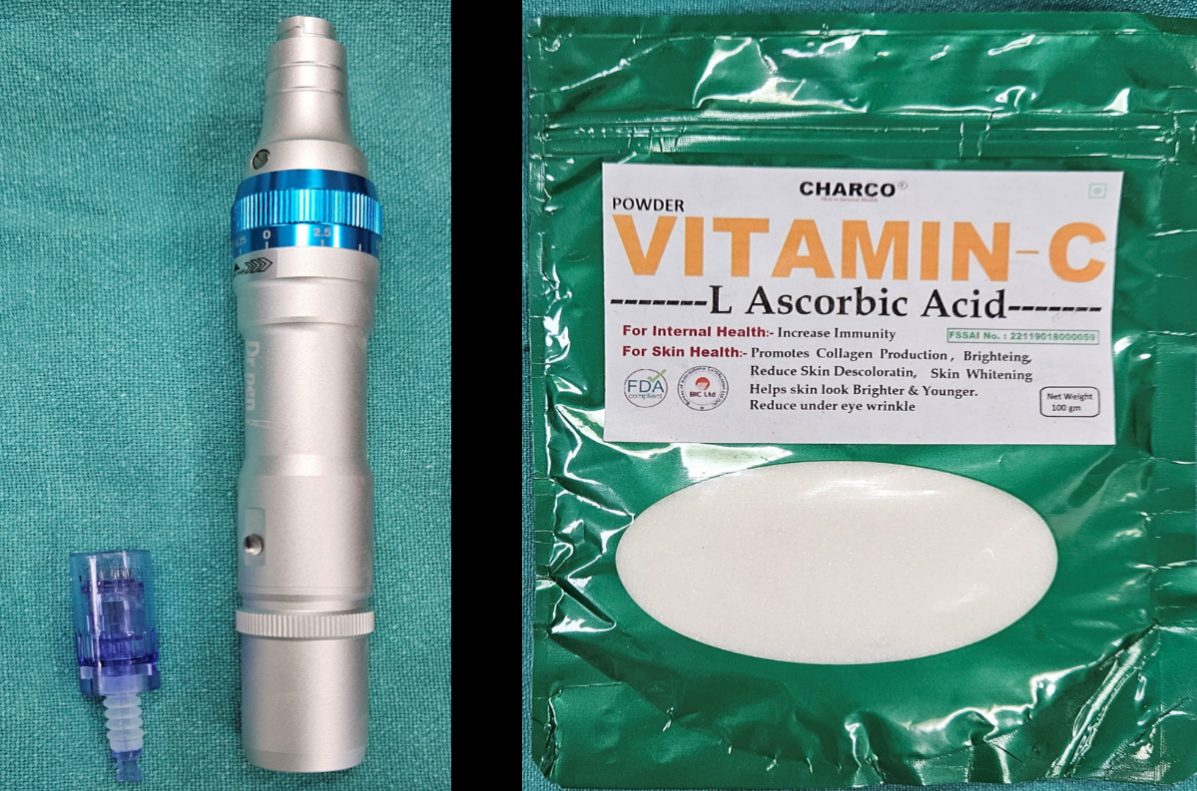
A) Derma pen. B) Topical vitamin C (1000 mg)

 In the test group, the MN technique was used. A Derma pen (Dr. Pen- ultima A6), an instrument resembling a pen with a handle, and rechargeable battery-powered needles, were used (Figure 3). The needle tips feature 12‒24 needles arranged in rows. The handpiece allows versatile treatment directions, adjustable needle length with guides, and disposable needle tips, facilitating use on different patients with the same autoclavable handpiece.

**Figure 4:**
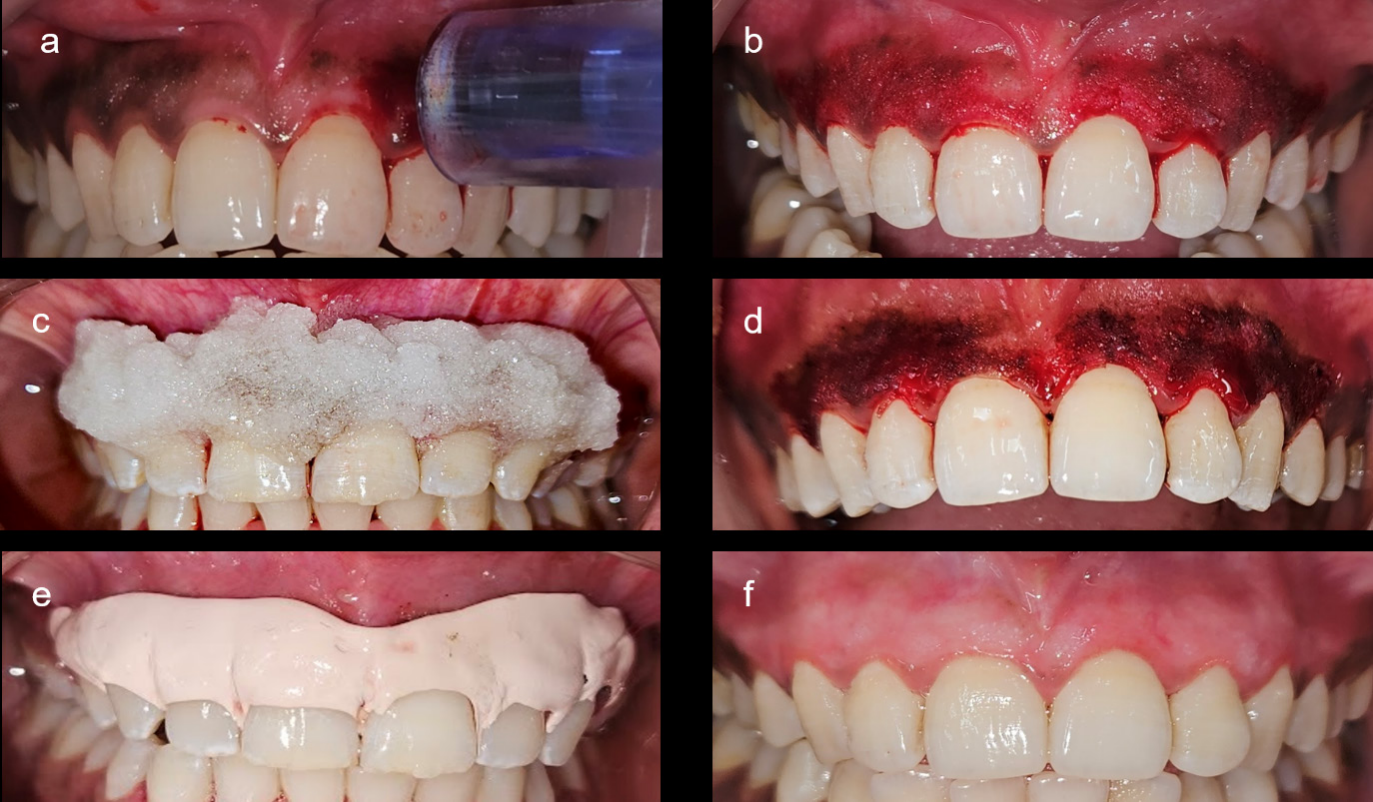
Depigmentation using microneedling with ascorbic acid. A) Bleeding points were created using a Derma pen. B) Ascorbic acid was applied for 10 min. C) Intraoperative view immediately after removing ascorbic acid. E) Coe-pack placed. F) Postoperative view after 1 month

 The region was anesthetized using 2% lignocaine hydrochloride. MN was done using a Derma pen up to a depth of 1.5 mm. When bleeding points were evident on the affected gingival epithelium, topical AA (CHARCO, 1000 mg/mL) mixed with saline was applied to the gingival mucosa for 10 minutes. AA was removed from the gingiva after 10 minutes, and a periodontal dressing was placed (Figure 4). The MN procedure was repeated at 2-week intervals.

**Figure 5:**
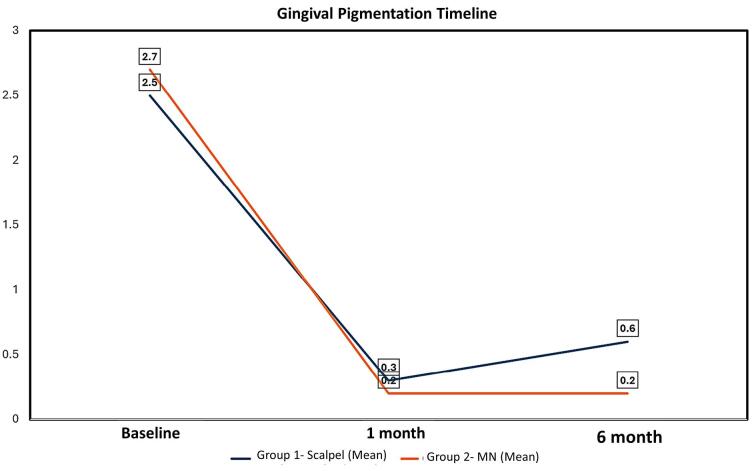
Intergroup comparison of depigmentation at baseline, 1 month, and 6 months. A higher rate of recurrence was observed with the scalpel technique

 The patients were advised to abstain from consuming acidic or hot beverages for 24 hours and to refrain from brushing over the surgical site for one week to prevent any mechanical trauma to the gingiva. In both groups, periodontal dressing was removed after 7 days, the patients were recalled at 1 and 6 months, and the following parameters, namely DOPI and HMI, were recorded by the same examiner (Figure 5).

###  Statistical analysis

 The data underwent statistical analysis using SPSS 23 for Windows (SPSS Inc., Chicago, Illinois, USA). Kruskal-Wallis test was conducted for ordinal variables to compare the results at baseline, 1 month, and 6 months, while categorical data underwent analysis using the chi-squared test. The results were presented as frequency tables, and a significance level of *P* < 0.05 was deemed statistically significant.

## Results

 The present study was designed to compare the two procedures of depigmentation, i.e., scalpel technique and MN, in 20 patients. There were no dropouts or postoperative complications in any treated cases.

 Intergroup comparison of bleeding during surgery showed significantly higher bleeding scores in the scalpel technique compared to MN with a *P*< 0.001, as shown in Table 1.

**Table 1:** Intergroup comparison of bleeding during surgery within the quadrants operated with different techniques

**Group**	**Patients with score 1**	**Patients with score 2**	**Patients with score 3**	**Patients with score 4**	**Total**	***P*** ** value**
ST	0	1	9	0	10	< 0.001*
MN	0	10	0	0	10	

ST: scalpel technique, MN: microneedling with ascorbic acid. *Statistically significant

 The postoperative pain score was evaluated on day 1 postoperatively. Statistical analysis revealed a significant association between pain and treatment techniques, with MN showing significantly low postoperative pain scores with a *P* = 0.008 (Table 2).

**Table 2:** Intergroup comparison of pain evaluated at 1 day postoperatively within the quadrants operated with different techniques

**Group**	**Patients with no pain**	**Patients with mild pain**	**Patients with moderate pain**	**Patients with severe pain**	**Total**	***P*** ** value**
ST	0	2	7	1	10	0.008*
MN	6	3	1	0	10	

ST: scalpel technique, MN: microneedling with ascorbic acid. *Statistically significant

 Wound healing was assessed at 7 days postoperatively, which revealed a statistically significant difference between the two groups (*P*< 0.001). The scalpel technique showed better wound healing after surgery compared to MN (Table 3).

**Table 3:** Intergroup comparison of wound healing at 7 days postoperatively within the quadrants operated with different techniques

**Group**	**Patients with very poor healing**	**Patients with poor healing**	**Patients with good healing**	**Patients with very good healing**	**Patients with excellent healing**	***P*** ** value**
Scalpel	0	0	6	4	0	< 0.001*
MN	0	3	6	1	0	

ST: scalpel technique, MN: microneedling with ascorbic acid *Statistically significant

 The intensity of gingival pigmentation at baseline, 1 month, and 6 months was assessed using the DOPI (Table 4 and Figure 5). There was a notable decrease in the intensity of pigmentation in both groups at 1 month compared to the baseline (*P* < 0.001). However, at six months, the DOPI value in the scalpel group increased to 0.60 ± 0.68 compared with the MN group (0.20 ± 0.410), which was significantly greater with a P-value of 0.03, suggesting the recurrence of gingival pigmentation with scalpel technique at 6 months. Similarly, the distribution of gingival pigmentation at baseline, 1 month, and 6 months was assessed using the HMI (Table 5). There was a statistically significant decrease in the distribution of melanin pigmentation in both groups at one month compared to the baseline (*P* < 0.001). However, HMI scores for the scalpel technique at six months increased to 0.45 ± 0.510, whereas MN scores remained the same (0.20 ± 0.410). The difference between these two groups was statistically significant, suggesting the recurrence of gingival pigmentation.

**Table 4:** Intergroup comparison of the intensity of gingival pigmentation at baseline, 1 month, and 6 months assessed using the Dummett oral pigmentation index

**Gingival pigmentation timeline**	**Group 1-ST** **(Mean±SD)**	**Group 2-MN** **(Mean±SD)**	***P*** ** value**
Baseline	2.50 ± 0.115	2.70 ± 0.470	0.206
1 month	0.30 ± 0.47	0.20 ± 0.410	0.478
6 months	0.60 ± 0.681	0.20 ± 0.410	0.03*
*P* value (baseline vs. 1 month)	< 0.001*	< 0.001*	
*P* value (baseline vs. 6 months)	< 0.001*	< 0.001*	

ST: scalpel technique, MN: microneedling with ascorbic acid, SD: standard deviation. *Statistically significant

**Table 5:** Intergroup comparison of the distribution of gingival pigmentation at baseline, 1 month, and 6 months was assessed using the Hedin melanin index

**Gingival pigmentation** **timeline**	**Group 1-ST** **Mean±SD**	**Group 2- MN** **Mean±SD**	***P*** ** value**
Baseline	3.60 ± 0.503	3.60 ± 0.503	0.801
1 month	0.35 ± 0.489	0.20 ± 0.410	0.302
6 months	0.45 ± 0.510	0.20 ± 0.410	0.046*
*P* value (baseline vs. 1 month)	< 0.001*	< 0.001*	
*P* value (baseline vs. 6 month)	< 0.001*	< 0.001*	

ST: scalpel technique, MN: microneedling with ascorbic acid, SD: standard deviation. *Statistically significant

## Discussion

 Gingival hyperpigmentation appears as a purplish, diffuse dark brown to black color on the gingiva, resulting from the deposition of melanin pigments by active melanocytes in the basal and spinous cell layers of the gingival epithelium. The enzyme tyrosinase plays a pivotal role in converting tyrosine into dopaquinone, a melanin precursor, through oxidation.^14^ Various surgical techniques, including scalpel surgery, electrosurgery, cryosurgery, bur abrasion, lasers, and radiosurgery, have been used to remove these pigmentations for esthetic purposes. Additionally, various graft materials such as acellular dermal matrix allografts and free gingival grafts are employed to conceal gingival pigmentation.^15^

 The scalpel surgical technique remains a pioneering and widely favored method due to its straightforward application, ready availability of equipment, and cost-effectiveness. This procedure entails surgical removal of the gingival epithelium and a layer of the underlying connective tissue, enabling the exposed connective tissue to heal through secondary intention, contributing to postoperative pain and patient discomfort.

 In the present study, gingival depigmentation was achieved through an innovative approach employing MN and topical application of AA. This method is minimally invasive, well-tolerated, safe, cost-effective, and requires less time.

 During the procedure, the precise bleeding points were created by MN, which was necessary to achieve optimal results.^16^ These micro-perforations facilitate the effective penetration of therapeutic medication into the connective tissues, stimulating the production of new collagen and impacting melanocytes.^17^

 Then, we applied topical AA as a therapeutic agent known for its efficacy in directly decreasing melanogenesis, thereby promoting depigmentation because melanin serves as a repository for reactive oxygen species (ROS), copper (Cu), and calcium (Ca) within cells. Once AA enters the targeted tissue, it interacts with melanin, depleting ROS, Cu, and Ca, consequently diminishing melanin synthesis.^18^ This highlights AA’s influence on melanocyte function rather than quantity, in contrast to alternative methods that involve melanocyte destruction and removal (scalpel method).

 The study found that MN had distinct advantages over the scalpel technique in terms of pain and bleeding but faced challenges regarding wound healing. MN resulted in significantly less postoperative pain, which can be attributed to its minimally invasive approach that causes micro-injuries without extensive tissue removal, reducing trauma to the gingival tissue. It also led to less bleeding, as the fine needles used in MN create controlled micro-injuries that minimally disrupt blood vessels. However, wound healing was less favorable with MN compared to the scalpel technique, as the micro-injuries were not as effective in fully removing pigmented tissue, potentially leading to prolonged inflammation and delayed healing. Despite these limitations, the DOPI and HMI indicated that MN achieved a more uniform and esthetically pleasing depigmentation, with statistically significant improvements over the scalpel technique, due to its ability to provide a smoother gingival appearance.

 Our findings demonstrated remarkable esthetic improvements, consistent with those reported by Yussif et al,^19^ in 2019, where intraepithelial injections of 1‒1.5 mL of AA (200‒300 mg) were used for the gingival depigmentation procedure. Their study highlighted the efficacy of direct AA delivery in reducing pigmentation incidence scores and area. Additionally, they comparedinjectable AA with conventional scalpel depigmentation procedure, concluding that AA injection into pigmented cells yielded outcomes comparable to those achieved through conventional surgery.

 Furthermore, our findings were consistent with the results of a study by Mostafa and Alotaibi,^20^ where topical vitamin C was applied to the gingiva after MN; they concluded that AA inhibited melanin pigmentation, resulting in complete gingival depigmentation that lasted for six months.

 In contrast, El-Mofty et al^21^ conducted a randomized comparative study involving 20 patients, with one group receiving intra-mucosal injections of AA and the other group receiving topically applied AA gel. Their findings indicated a significant reduction in the mean area fraction of melanin-forming cells in both groups. However, the effect size was smaller in the group treated with topically applied AA gel (correlation coefficient *r* = 0.886) compared to intra-mucosal injections (*r* = 0.797).

 Additionally, our observations align with Shimada et al,^7^ who administered topical vitamin C gel to the gingiva and determined that AA inhibited melanin pigmentation.

 The findings of the current study indicated that both techniques yielded statistically comparable results. However, certain clinical observations attract our attention:

1. Both techniques were efficacious in reducing the melanin pigmentation; however, at 6 months, pigmentation tended to recur in the scalpel group, suggesting long-term superior esthetic results with the MN technique.
2. Individuals with highly pigmented gingiva should avoid undergoing MN with topical AA, as multiple sessions are required.
3. MN with topical AA treatment resulted in minimal pain scores.
4. Control over penetration depth is not achievable with MN with topical AA.
5. The requirement for multiple appointments and punctures in MN with topical AA may pose a disadvantage regarding patient compliance and wound healing.

## Conclusion

 This study compared two techniques for gingival depigmentation. Being a gold standard, the scalpel technique offers advantages, which include better patient compliance and excellent wound healing; it also has disadvantages like more bleeding and pain scores. Conversely, the MN technique emerges as an advanced technique, demonstrating less pain and bleeding and stable results after the gingival depigmentation procedure. Within the limitations of the study, it can be concluded that the MN technique is superior in terms of enhancing patient comfort and overall satisfaction, aligning with the evolving preferences for minimally invasive dental procedures. However, further randomized control studies with larger sample sizes and extended follow-up periods are required for more concrete evidence.

## Competing Interests

 The authors declare no conflicts of interest in this work.

## Ethical Approval

 The present study was conducted according to the Declaration of Helsinki and approved by the Ethics Committee of Maulana Azad Institute of Dental Sciences under the code ECR/1799/inst /DEL/2023. The authors certify that the study was performed in accordance with the relevant guidelines and regulations.
